# Concordance analysis between dosed serum bicarbonate and that calculated by gas analysis in chronic renal patients

**DOI:** 10.1590/2175-8239-JBN-2019-0236

**Published:** 2020-05-11

**Authors:** Luis Gustavo Modelli de Andrade, Ananda Barbosa Muniz, Alessandro Lia Mondelli, Daniela Ponce

**Affiliations:** 1 Universidade Estadual Paulista, Departamento de Medicina Interna, São Paulo, SP, Brasil.

**Keywords:** Renal Dialysis, Bicarbonates, Blood Gas Analysis, Diálise Renal, Bicarbonatos, Gasometria

## Abstract

**Introduction:**

The control of metabolic acidosis in dialysis patients focuses on the supply of bicarbonate during the dialysis session, and it is not standard in all hemodialysis to assess serum bicarbonate concentrations. Bicarbonate expressed in blood gas analysis is the most sensitive standard of analysis and it is measured indirectly, using the Henderson-Hasselbalch equation. There are no studies in this population evaluating the concordance between the calculated bicarbonate with the direct method of biochemical analysis. The aim of this study was to analyze the concordance between the measured and calculated serum bicarbonate levels using blood gas analysis.

**Methods:**

We analyzed blood samples from chronic kidney patients undergoing hemodialysis, using the same sample of bicarbonate analysis by biochemistry and gasometry. The concordance was assessed using the Bland-Altman method.

**Results:**

51 samples were analyzed. The analysis revealed a high correlation (r = 0.73) and a mean difference (bias) of 1.15 ± 3 mmol/L. The median time between collection and examination was 241 minutes.

**Discussion:**

We can conclude that the biochemical bicarbonate analysis compared to that calculated from blood gas analysis in chronic renal patients was consistent. For greater concordance between the data, it is important that the time between the collection of the samples and the referral to the laboratory for carrying out the dosages does not exceed four hours. The serum bicarbonate dosage can result in cost savings when compared to that of bicarbonate in blood gas analysis.

## Resumo

**Introdução:**

O controle da acidose metabólica em pacientes dialíticos está voltado, principalmente, para o suprimento de bicarbonato durante a sessão de diálise, não sendo padrão em todas as hemodiálises avaliar as concentrações séricas do bicarbonato. O bicarbonato expresso na gasometria é considerado o padrão mais sensível de análise e é medido indiretamente por meio da equação de Henderson-Hasselbalch. Não há estudos nessa população avaliando a concordância do bicarbonato calculado com o método direto de análise bioquímica. O objetivo deste estudo é analisar a concordância entre o bicarbonato sérico medido e o calculado por meio da gasometria.

**Métodos:**

Foram analisadas amostras de sangue de pacientes renais crônicos em hemodiálise sendo feito na mesma amostra de análise do bicarbonato pela bioquímica e análise pela gasometria. A concordância foi avaliada pelo método de Bland-Altman.

**Resultados:**

Foram analisados um total de 51 amostras. A análise de correlação revelou alta correlação (r = 0.73) e a diferença média (bias) de 1.15 ± 3 mmol/L. O tempo mediano entre a realização da coleta e do exame foi de 241 minutos.

**Discussão:**

Podemos concluir que a realização da dosagem bioquímica do bicarbonato comparada com a calculada a partir da gasometria em pacientes renais crônicos foi concordante. Para maior concordância entre os dados, é importante que o tempo entre a coleta das amostras e o encaminhamento ao laboratório para a realização das dosagens não exceda quatro horas. A dosagem do bicarbonato sérico pode resultar numa economia de custos comparada à do bicarbonato da gasometria.

## Introduction

Metabolic acidosis is highly prevalent in dialysis patients, and it is associated with changes in protein and glucose metabolism, bone and muscle diseases, cardiovascular diseases and increased mortality. The real prevalence of this problem in Brazil is unknown, since, in 1996, the dialysis regulatory agency published a decree suspending the mandatory measurement of bicarbonate in patients undergoing renal replacement therapy (RRT). The most recent public guidelines recommend that bicarbonate be measured every six months in patients with stage 4 CKD or quarterly in those with stage 5 and under conservative treatment; keeping the measurement of this parameter as not mandatory for patients on RRT.^1^^-^ 4

Currently, the control of metabolic acidosis in dialysis patients focuses mainly on the supply of bicarbonate during the dialysis session; however, we still need more studies to define the target serum bicarbonate level and the best dialysate bicarbonate concentration.

The bicarbonate expressed in gasometry is considered the most sensitive standard of analysis and it is not directly measured, but calculated by the Henderson-Hasselbalch equation, using the measured pH and partial pressure of carbon dioxide (PaCO2) values.^5^ The direct method of biochemical analysis has shown concordant results in some studies,^6^^,^ 7 and discordant in others.^5^^,^ 8 The economic impact of the serum bicarbonate analysis favors the performance of the procedure at a cost up to 100 times lower than that of bicarbonate calculated by gasometry. There are no concordance studies between the two analytical methodologies for the population of chronic renal patients.

The goal of this study was to analyze the concordance between the measured serum bicarbonate and its value calculated by gasometry; and to discuss which are the best methods to evaluate the agreement between the different tests.

## Materials and methods

We analyzed blood samples from chronic kidney patients undergoing hemodialysis using the same bicarbonate analysis sample by biochemistry and blood gas analysis. Chronic renal patients referred for kidney transplantation at the time of the biochemical compatibility tests participated in the study, with an additional collection of blood gases and biochemical analyses. We collected the samples in the period off hemodialysis, on Wednesdays or Thursdays, to avoid longer intervals without dialyses.

We recorded the time until the exams were processed from collection to processing. The Research Ethics Committee of the School of Medicine of Botucatu, approved this investigation, under number CAE: 23889019.4.0000.5411

We measured the serum bicarbonate using the VITROS^®^ 5.1|FS system from Ortho Clinical Diagnostics, which uses MicroSlide VITROS^®^ - dry chemistry technology. The ECO2 VITROS Slide has five layers, covered by a polyester layer. We add a drop of biological material to the slide, where it is evenly distributed to the adjacent layers. In the final reaction, oxaloacetate NADH oxidation and reduction produces NAD+ and malate. The slide needs to be incubated at 37ºC and we establish the CO_2_ concentration in the sample measuring the absorbance of the NADH that did not participate in the reaction through reflectance spectrophotometry.^9^ The samples in dry tubes with gel containing the patients’ serum were placed in the equipment as soon as they arrived at the laboratory, and we recorded their respective results and collection time.

We calculated the bicarbonate dosage through gasometry, using the Nova Biomedical Start Profile Prime equipment. This equipment combines microelectronics with the MicroSensor Card^TM^ in a blood gas analyzer.^10^ The samples in 1 ml syringes with heparin were homogenized and placed in the equipment as soon as they arrived at the laboratory. We recorded their respective results and collection time.

### Statistical analysis

To calculate the sample number we used the method proposed by Lu MJ et al.^11^ We considered an initial pilot sample with 10 cases. In these cases, we obtained an average difference between the methods divided by the standard deviation of 2.9. Considering an alpha of 0.05 and beta of 0.80. We had a sample of 48 cases.

We performed the analysis using the Pearson’s correlation between the two dosages. To assess agreement, we used the Bland-Altman method,^12^ which is based on the average difference between the two dosages, which should be in the range of 2 times the standard deviation (upper threshold and lower threshold). We used the R version 3.4.2 software.

## Results

**Figure 1:**
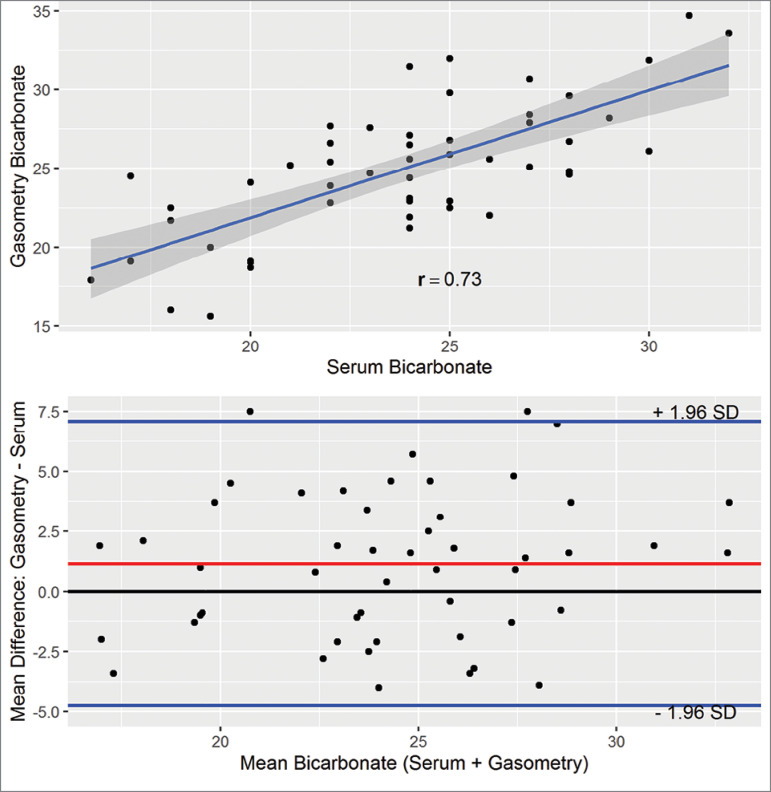
Correlation between serum bicarbonate and gasometry bicarbonate. Linear regression with r = 0.73. Bland-Altman diagram showing the mean difference on the y-axis by the mean of the two groups. The red line represents the mean difference (Bias) and the blue lines, the upper and lower concordance thresholds.

We analyzed 51 samples, and our analysis revealed a high correlation (r = 0.73, *p* < 0.001), with a mean difference (bias) of 1.15 ± 3 mmol/L (Table 1, Figure 1).

**Table 1:** Concordance and correlation among the biochemical and gas analysis data

Number of samples	51
Mean difference (Bias)	1.15
Upper concordance threshold (+1.96 x dp)	7.07
Lower concordance threshold (-1.96 x dp)	-4.75
Critical difference	5.91
Pearson's correlation (r)	0.728
Correlation p-value	< 0.001

**Figure 2:**
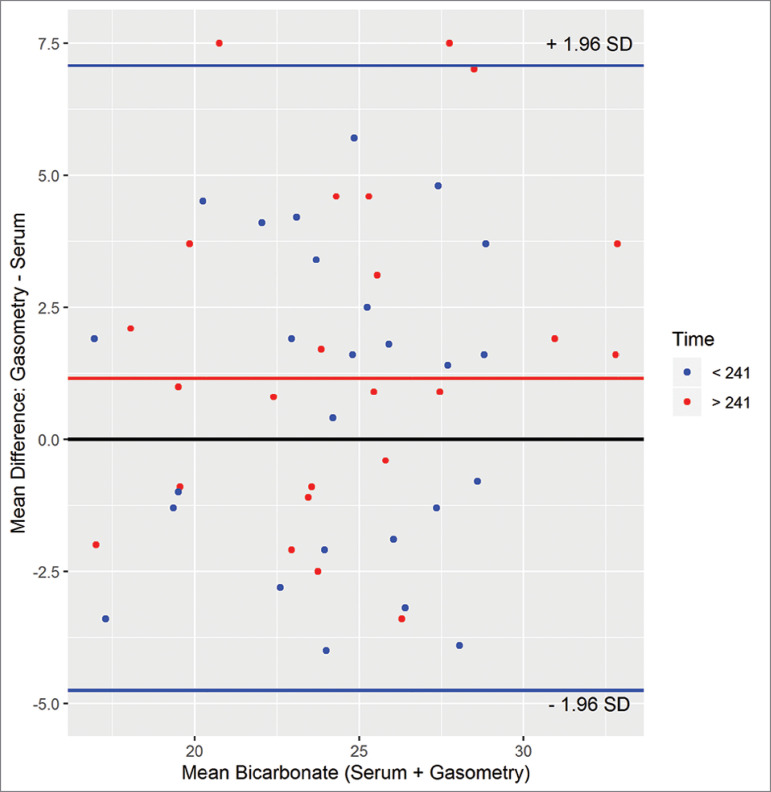
Bland-Altman diagram showing the mean difference on the y-axis by the mean of the two groups. The red line represents the mean difference (Bias) and the blue lines, the upper and lower concordance thresholds. The colors of the dots represent the median collection time: higher or lower than 241 minutes.

The median time between collection and examination was 241 minutes. There was no correlation between the collection time and the average bicarbonate difference (r = 0.14 and *p* = 0.32); however, the cases that showed the greatest mean difference (bias) were those analyzed after the median period of 241 minutes (Figure 2).

## Discussion

In this study, we evaluated the concordance between the values of bicarbonate calculated by gasometry and those measured by biochemistry, in stage-5 chronic kidney patients. We found a median correlation between the dosages with a relative concordance between the measurements. The mean difference (bias) was 1.15 mmol/liter, and when we evaluated the Bland-Altman diagram, most cases were between 1.98 of standard deviation.^12^ The largest differences (greater than 7 mmol/liter) were found in cases where the interval between the collection and the examination was greater than 240 minutes. When analyzing only samples in which the period between collection and dosage was less than 4 hours, the average difference between the methods was 1.15 mmol/liter, considered clinically and statistically acceptable, since most of the differences are in the confidence interval.

Considering the routine of a hemodialysis unit, the four-hour time between collection and exams is acceptable. In this context, the concordance between the bicarbonate values calculated by gasometry and those measured by biochemistry assessed by the Bland-Altman diagram was in the desirable range of up to two standard deviations.

The use of correlation alone is not a good parameter to assess the concordance between the two tests, as previously discussed by Bland and Altman,^12^ who proposed a method based on the average difference between the two measures. Considering that the average difference follows a normal distribution, the difference between the two methods (bicarbonate calculated from gasometry and that measured by biochemistry) must be between 1.98 standard deviation, at the lower and upper thresholds. Placing the data on a graph, we observe the mean difference (y-axis) by the mean of the results (mean of the gas analysis and biochemistry bicarbonate) on the x-axis. Two more lines are drawn with the confidence intervals. The upper line comprises the value of the average added to 1.98 times the standard deviation and the lower line, the average minus 1.98 times the standard deviation. It is expected that the mean differences are situated close to the reference line of zero and between the two threshold lines of the standard deviations.^13^ Thus; one can evaluate the concordance between the two tests more appropriately by visual inspection of the Bland-Altman chart.

Possible limitations of this study reside in its unicentric nature, limiting its reproducibility, where different techniques of biochemical analysis or gas analysis are used. Measurements were made in hemodialysis patients, limiting their extrapolation to pre-dialysis or peritoneal dialysis patients.

We can conclude that the biochemical dosage of bicarbonate compared to that calculated from blood gas analysis in chronic renal patients is consistent. At HC UNESP, after this concordance analysis, it was possible to realize cost savings in bicarbonate dosages, especially in the routine of patients undergoing chronic hemodialysis therapy/ which can be extended to the entire country. Considering a dialysis with 200 patients and an average cost of R $ 20.00 reals for blood gas analysis, and R $ 0.30 cents for serum bicarbonate, the monthly cost of bicarbonate dosage by blood gas analysis is R $ 4,000.00 compared to R $ 60.00 reals for biochemistry. For greater concordance among the data, it is important that the time between the collection of samples and the referral to the laboratory for the performance of the dosages does not exceed four hours.

## References

[1] Yamamoto T, Shoji S, Yamakawa T, Wada A, Suzuki K, Iseki K (2015). Predialysis and Postdialysis pH and Bicarbonate and Risk of All-Cause and Cardiovascular Mortality in Long-term Hemodialysis Patients. Am J Kidney Dis.

[2] Tentori F, Karaboyas A, Robinson BM, Morgenstern H, Zhang J, Sen A (2013). Association of dialysate bicarbonate concentration with mortality in the Dialysis Outcomes and Practice Patterns Study (DOPPS). Am J Kidney Dis.

[3] Brasil, Ministério da Saúde, Portaria nº 2.042, de 11 de outubro de 1996 (1996). Estabelece o Regulamento Téc-nico para o funcionamento dos Serviços de Terapia Renal Substitutiva e as normas para cadastramento des-ses estabelecimentos junto ao Sistema Único de Saúde. Ministério da Saúde.

[4] Brasil, Ministério da Saúde (2014). Diretrizes Clínicas Para o Cuidado ao Paciente com Doença Renal Crônica - DRC no Sistema Único de Saúde.

[5] Mohd Nasir N, Sthaneshwar P, Megat Yunus PJ, Yap SF (2010). Comparing measured total carbon dioxide and calculated bicarbonate. Malays J Pathol.

[6] Kumar V KB (2008). Comparison of measured and calculated bicarbonate values. Clin Chem.

[7] Chittamma A, Vanavanan S (2008). Comparative study of calculated and measured total carbon dioxide. Clin Chem Lab Med.

[8] Story DA, Poustie S (2000). Agreement between two plasma bicarbonate assays in critically ill patients. Anaesth Intensive Care.

[9] ORTHOCLINICALDIAGNOSTICS https://www.orthoclinicaldiagnostics.com/pt-br/home.

[10] NOVABIO https://www.novabio.us/pt/home.php.

[11] Lu MJ, Zhong WH, Liu YX, Miao HZ, Li YC, Ji MH (2016). Sample Size for Assessing Agreement between Two Methods of Measurement by Bland-Altman Method. Int J Biostat.

[12] Bland JM AD (1986). Statistical methods for assessing agreement between two methods of clinical measurement. Lancet.

[13] Nawarathna LS, Choudhary PK (2013). Measuring agreement in method comparison studies with heteroscedas-tic measurements. Stat Med.

